# Is Delayed Pressure Urticaria Associated with Increased Systemic Release of sCD40L?

**DOI:** 10.1155/2013/823798

**Published:** 2013-10-23

**Authors:** T. Jasinska, A. Grzanka, E. Machura, A. Kasperska-Zajac

**Affiliations:** ^1^Chair and Clinical Department of Internal Diseases, Dermatology and Allergology ul. M. Curie-Skłodowskiej 10, Medical University of Silesia in Katowice, 41-800 Zabrze, Poland; ^2^Department of Pediatric in Zabrze, Medical University of Silesia, Poland

## Abstract

*Background*. Elevated levels of soluble CD40 Ligand (sCD40L) were found in serum but not in plasma of patients with chronic spontaneous urticaria (CU). What is important is that sCD40L has proinflammatory properties, and its elevated plasma level may indicate increased risk of cardiovascular events. These observations should stimulate further evaluation of sCD40L in different forms of urticaria. *Aim*. In the present study, sCD40L plasma level was investigated in delayed pressure urticaria (DPU). *Methods*. As platelets are predominant and variable sources of sCD40L, we investigated sCD40L concentration in platelet-poor plasma (PPP), which seems the best way to minimize the potential contribution of these cells to the ligand level. *Results*. Plasma sCD40L concentration was significantly increased in the DPU group compared to the healthy controls. *Conclusions*. It seems that DPU is associated with increased systemic release of sCD40L, which is believed to derive predominantly from activated platelets. The present study as well as the earlier contributions suggest that distinct cells activity, including platelets, may be identified in different types of urticaria.

## 1. Introduction

Chronic spontaneous urticaria (CU) and delayed pressure urticaria (DPU) are associated with systemic inflammatory reaction that is called acute-phase response (APR) as well as other signs of inflammatory activity [1–4].

It has been documented that platelets contribute to immune-inflammatory processes [5, 6]. However, data regarding behaviour of these cells and their mediators in different forms of urticaria are very scarce. 

CD40L is the transmembrane protein of the tumor necrosis factor family [7] expressed by different cell types, including lymphocytes [8], mast cells, basophils [9], and platelets [10, 11], and it is subsequently cleaved generating a circulating soluble form of CD40L (sCD40L). Circulating sCD40L is believed to derive predominantly from activated platelets and therefore may reflect platelet activation [10, 11]. 

Elevated circulating levels of sCD40L have been observed in a variety of inflammatory diseases where sCD40L is thought to promote proinflammatory effects [12–14]. 

It has been demonstrated that serum sCD40L concentration is significantly increased in CU patients as compared to healthy subjects, suggesting that sCD40L may be involved in immune activation observed in this disease [15, 16]. On the contrary, we did not find significant differences in sCD40L concentration in platelet-poor plasma (PPP) between CU patients and the healthy subjects, indicating that free circulating sCD40L may not be elevated in CU patients [17]. Taken together, this may confirm that serum may be better to assess a total pool of sCD40L stored in platelets and other cells; on the other hand, PPP is more useful to measure free circulating levels of sCD40L in vivo [18, 19].

Taking into account both DPU as a distinct form of urticaria and the contribution of platelets to inflammation in DPU, it seems interesting to investigate release reaction of sCD40L in the disease. In the present study, sCD40L plasma level was investigated in DPU.

## 2. Methods

Eighteen symptomatic patients (11 men and 7 women) of age range 26–44 years (the median age 37 years), nine patients with pure DPU (DPU alone subgroup) and nine patients with concomitant chronic spontaneous urticaria (DPU/CU subgroup), were enrolled into the study. All identified causes of urticaria, including autoreactivity and concomitant diseases had been excluded. Seven patients suffered from severe DPU, urticarial lesions still elicited even under slight pressure. In the remaining 11, less extensive urticarial lesions were observed during physical examination.

At least three weeks before the examination, the patients with severe disease ceased to take corticosteroids (except for 1 patient). The antihistamines were withdrawn 3-4 days before the study. 

The control group consisted of 27 nonsmoking healthy patients (12 females and 15 males; the median age was 36 years, range 27–43) without any medication. 

The Local Ethics Commission approved this study, and written informed consent was obtained from all participating subjects.

### 2.1. Blood Sampling and Plasma CD40L Measurement

Whole blood was obtained in the morning (7.00 a.m. to 8.00 a.m., in the fasting state) after 25-minute rest at slight or no stasis from an antecubital vein into EDTA (theophylline, adenosine, and dipyridamole) tubes then immediately placed in melting ice. For platelet-poor plasma (PPP) preparation the samples were centrifuged at 1000 ×g for 15 minutes at 4°C, followed by an additional centrifugation at 10000 ×g for 10 min to ensure elimination of platelets. PPP samples were stored at –80°C until analysis. Quantification of sCD40L was performed with a commercial enzyme-linked immunosorbent assay (ELISA) kit (R&D System, Abingdon, United Kingdom) according to the manufacturer's instructions. 

Each sample was measured in duplicate. At our laboratory, the intra-assay and interassay coefficients of variation were <8%. 

The number of peripheral blood platelets was measured by the automated haematology analyser.

### 2.2. Statistical Analysis

Data are presented as median and quartile ranges. Kruskal-Wallis variance analysis was used for screening differences between the groups. Mann-Whitney *U* test was used to compare data between the patient groups and the healthy controls. Spearman's rank test was performed for correlations. *P* values below 0.05 were considered significant.

## 3. Results

**Figure 1:**
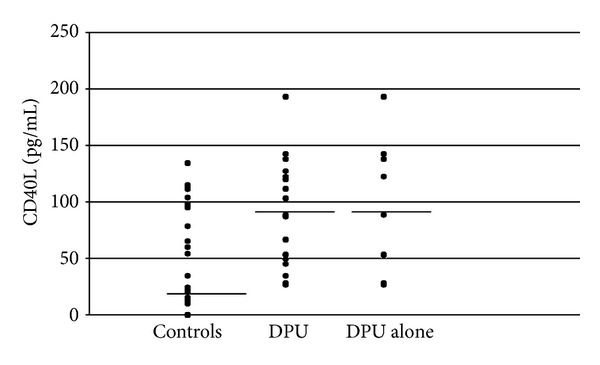
Individual plasma sCD40L concentration in delayed pressure urticaria (DPU) patients in comparison to healthy subjects. Horizontal lines represent median value. Plasma sCD40L concentration in the control group was significantly higher as compared with DPU as a whole and DPU without concomitant CU (DPU alone); *P* = 0.002 and *P* = 0.013, respectively.

Plasma sCD40L concentrations were significantly higher in DPU as a whole and DPU without concomitant CU (DPU alone) as compared with the healthy subjects (median (quartile range): 87.9 (49.3–122.4), 88.6 (53.1–138.2), and 24.2 (12.2–93.3) pg/mL, resp.) (Figure 1). No significant differences were found between the three groups in platelet count (data not shown).

No significant correlations were observed between CD40L concentrations and platelet count and CRP concentration (*r* = 0.13, *P* = 0.60 and *r* = −0.16, *P* = 0.53, resp.).

## 4. Discussion

To the best of our knowledge, this study is the first to provide important information regarding increased plasma sCD40L levels in DPU. However, data regarding behaviour of these cells and their mediators in different forms of urticaria are very scarce [20, 21]. 

Elevated serum level of soluble sCD40L was found in patients with CU, suggesting that in this disease sCD40L may be involved in immune activation [15, 16]. On the other hand, we have not found any significant differences in plasma sCD40L levels in CU patients [17]. Taken together, the results suggests that total pool of sCD40L stored in platelets and other cells is increased in CU, but free circulating sCD40L is not elevated.Contrary to the previous results, we observed increased plasma concentration of sCD40L in DPU patients. The discrepancy of the results is unclear. Firstly, DPU is a distinct form of urticaria characterized by deep inflammatory infiltrate and systemic symptoms.

Secondly, changes in platelet activity have been reported in DPU indicating on enhanced platelet activity measured by PF-4 and beta-thromboglobulin plasma concentration [22]. The translocation of CD40L seems to coincide with the release of *α*-granule contents including platelet factor 4 (PF-4). In addition, it is estimated that 95% of the circulating sCD40L is derived from platelets. However, platelet may not be the sole source of sCD40L in DPU, and other cells may contribute to the increased circulating level [18, 19]. 

The mechanisms leading to platelet activation and/or increased sCD40L release in DPU were not identified in this study. Circulating platelets are highly reactive to exogenous and endogenous stimuli, including cytokines and immune mediators. Expression of CD40L is induced by proinflammatory stimuli, such as IL-3 and tumor necrosis factor-alpha (TNF-*α*), which are known to increase in DPU [23]. On the other hand, it seems that APR, manifested by increased concentration of CRP, does not play a direct role in upregulation of a transmembrane sCD40L release. On the one hand, platelet hyperactivity has not been observed in CU which is known to be associated with APR [17]. In addition, there was no correlation between sCD40L and CRP concentrations in DPU. 

CD40L or its circulating soluble counterpart (sCD40L) may upregulate inflammatory and immune responses [12–14]. Therefore, we speculate that the protein has proinflammatory activity in DPU and contributes to amplification of the urticarial inflammation.

What is important is that elevated plasma sCD40L and CRP concentrations may indicate the increased risk of cardiovascular events [24, 25]. The observations made recently and our results suggest an increased risk of cardiovascular events in severe and long-lasting DPU, which might result from increased concentration of these biomarkers. 

## 5. Limitation

Because of the small study sample, future research is needed to prove release of sCD40L from platelets and to get more insights into the mechanisms of platelet activation and sCD40L function in DPU.

## 6. Conclusions

Our results indicate that DPU is associated with increased circulating sCD40L level, which is believed to derive predominantly from activated platelets. These findings may confirm ongoing platelet activation in DPU, manifested by increased chemokines and sCD40L release into the circulation. The present study as well as the earlier contribution suggest that distinct cells activity, including platelets, may be identified in different types of urticaria, similarly to that reported in atopic diseases [26].
